# Comparative Analysis of Dentoalveolar Effects Induced by Various Fixed Functional Appliances: A Three-Dimensional Finite Element Study

**DOI:** 10.3390/jcm15156021

**Published:** 2026-08-03

**Authors:** Eyüp Burak Küçük, Mustafa Onur Şengezer

**Affiliations:** Department of Orthodontics, Faculty of Dentistry, Hatay Mustafa Kemal University, Hatay 31060, Türkiye; monursengezer@gmail.com

**Keywords:** fixed functional appliance, finite element analysis, Herbst appliance, Forsus FFRD, PowerScope2

## Abstract

**Background/Objectives:** The aim of this study was to quantify the dentoalveolar effects of three different orthodontic fixed functional appliances used to correct Class II Division 1 malocclusion using the finite element method. **Methods:** Three-dimensional models of the mandible, maxilla, and skull were generated from cone–beam computed tomography scans of a patient with Class II malocclusion. The PowerScope2, Forsus Fatigue Resistant Device (FFRD), and Herbst appliances were scanned, digitally reconstructed and positioned on simulated dental arches. Appliance forces were applied to the meshed models to create three different loading conditions. Von Mises and principal stresses in the dentoalveolar structures and instantaneous tooth displacements were calculated using the finite element method. **Results:** In all simulations, the maxillary teeth moved distally, whereas the mandibular teeth moved mesially, with the latter showing greater overall movement. The Herbst appliance produced the greatest maxillary distalization, while the PowerScope2 caused the greatest mandibular mesialization. Von Mises stress was mainly concentrated on the buccal surfaces, especially on the maxillary first molars, with appliance-specific increases in the premolars/canines and additional palatal stress in the Herbst simulation. Principal stress patterns were similar for the FFRD and PowerScope2, whereas the highest principal stress values were observed in the Herbst simulation. **Conclusions:** Within the specific boundary conditions of this patient-derived model, the Herbst simulation produced the highest stress values while inducing minimal initial mesial displacement in the mandible, theoretically indicating a potential for reduced mandibular dentoalveolar displacement. In addition, the Herbst appliance demonstrated a more pronounced initial distalizing effect on the maxillary dentition under these simulated conditions. However, due to the inherent reliance on specific static muscle force assumptions, these biomechanical trends should be interpreted with caution and require further dynamic and clinical validation before drawing definitive conclusions regarding clinical advantages.

## 1. Introduction

The prevalence of Class II malocclusion ranges between 1.6% and 63%, and the overall global prevalence is 19.5% [1]. According to McNamara, mandibular advancement has become a prominent treatment modality, as mandibular retrusion is the primary characteristic of Class II Division 1 malocclusion [2]. While various removable appliances have been used for the treatment of Class II malocclusion, fixed functional appliances (FFAs) provide distinct advantages, such as shortened treatment times and diminished risks associated with patient compliance [3].

Fixed functional appliances correct Class II malocclusion by stimulating mandibular growth, ensuring the distalization of maxillary teeth and the mesialization of mandibular teeth [4,5]. The Herbst appliance is a well-known rigid FFA that is effective in adults and growing Class II patients [6,7,8]. It can be used as an alternative to sagittal split osteotomy in adult patients [7,8,9]. The PowerScope and Forsus Fatigue Resistant Device (FFRD) are semi-rigid FFAs which lead to continuous force application to the dental arches via spring-loaded mechanisms. The FFRD is a widely used mandibular advancement spring. While it produces a marked skeletal response in growing patients, its effects are predominantly dentoalveolar in post-pubertal patients [10]. The newly developed PowerScope2 appliance corrects Class II malocclusion via a mechanism similar to that of the FFRD. Nevertheless, its standardized sizing and the elimination of the need for molar bands constitute notable advantages over the FFRD. Although the FFRD and PowerScope2 appliances are not as rigid as the Herbst, they are designed to apply continuous force to the mandible by spring-loaded mechanisms without limiting mastication and lateral jaw movements. Due to these inherent characteristics, these appliances enhance patient comfort and maintain durability.

The finite element method is used to simulate the clinical situations and to assess the stress, strain and movement during orthodontic treatment. The biomechanical effects of orthodontic forces can be evaluated by using this method [11]. It is possible to determine the physical properties and elasticity coefficient of living tissues after constructing the structures of these tissues in three dimensions [12,13]. This system is based on solving problems by using various software. It is a noninvasive technique. With this system, it is possible to see the direction of orthodontic movement, and there is no need for patient in such studies. Thus, problems regarding patient compliance, complications and long study periods can be eliminated.

There are various studies in which the treatment of Class II malocclusion has been evaluated by using the finite element method [11,12,13,14,15,16]. Nevertheless, most of these studies have assessed the effects of FFAs on the temporomandibular joint or masticatory muscles [11,13,15] rather than their effects on dentoalveolar tissues. Moreover, in these studies, earlier versions of software have been used, which makes it impossible to mimic a lifelike model because of the number of nodes and elements used in three-dimensional (3D) models [11,13,15]. While recent finite element studies have focused on dentoalveolar structures [17,18,19], there remains a notable gap in the literature regarding a direct biomechanical comparison of the most commonly used FFAs in daily clinical practice. Therefore, the aim of this study was to create lifelike 3D finite element models of the PowerScope2, Herbst and FFRD appliances, and to compare the effects of these appliances in terms of von Mises stress, principal stress and tooth displacement.

## 2. Materials and Methods

The study was approved by the Ethics Committee on Clinical Trials of Hatay Mustafa Kemal University (Approval No: 10, Date: 28 March 2019). The three-dimensional patient data used in this study were obtained from the archive of Ay Tasarım Ltd. Şti (Ankara, Türkiye), and all finite element analyses were performed by the same company. The 3D geometry of the edentulous maxilla, mandible and skull were reconstructed from the cone–beam computed tomography (CBCT) images taken from a young adult with skeletal Class II malocclusion. The device was operated at 120 kVp, 3.8 mA, with a 40 s scanning time. A total of 601 slices were attained for the entire skull. To obtain a convenient 3D model of the skull, CBCT images were processed (first interactive segmentation, then complex render) with 3D-Doctor software version 4.0 (Able Software Corp., Lexington, MA, USA).

Upon scanning the teeth, excluding the third molars, on the x, y, and z axes with a CBCT scanner, 3D dental arch models were constructed. The Rhinoceros version 4.0 CAD software (Robert McNeel & Associates, Seattle, WA, USA) was used to align them into a U-shaped arch form. Subsequently, they were mounted on the 3D upper and lower dental arch models as homogeneous structures without detailing the enamel, dentin or cementum layers [20]. The periodontal ligaments (PDL) and cortical bone layers were modelled around the roots with a thickness of 0.2 mm.

The 3M Unitec Gemini metal brackets (slot size: 0.022-inch; Monrovia, CA, USA), stainless steel arch wires (0.019 × 0.025-inch in size), first and second molar tubes (slot size: 0.022-inch), Forsus Fatigue Resistant Device (FFRD; 3M Unitek, Monrovia, CA, USA), Herbst (Dentaurum Inc., Newtown, PA, USA), and PowerScope2 (American Orthodontics, Sheboygan, WI, USA) appliances were scanned with a Smart Optics 3D scanner (Sensortechnik GmbH, Bochum, Germany), and the 3D models were generated in STL format.

**Figure 1:**
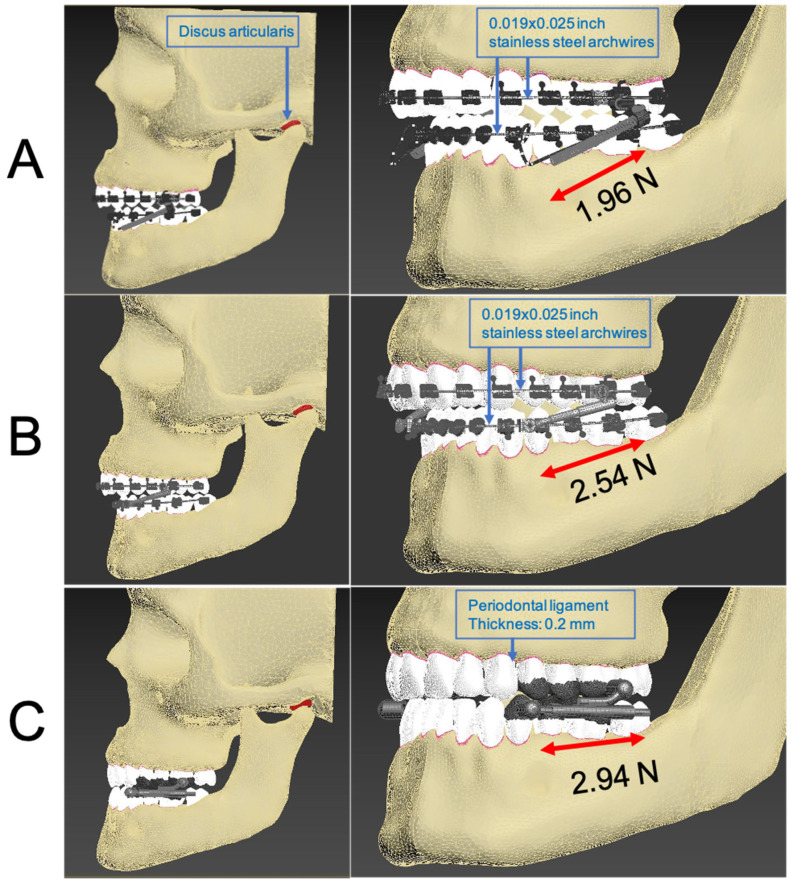
Meshed finite element models and the direction of the applied forces in a lateral view: (**A**) the FFRD simulation; (**B**) the PowerScope2 simulation; (**C**) the Herbst simulation (Note the origin of the force in the Herbst simulation. When the mandible moves forward due to the telescopic mechanism, the posterior fibers of the temporalis muscle stretch, and a retrusive force occurs on the mandible. The origin of the force is the coronoid process in this simulation).

Brackets and molar tubes were meticulously positioned at the ideal vertical and mesiodistal orientations on the tooth crowns, before the application of PowerScope2 and FFRD. Stainless steel arch wires of 0.019 × 0.025-inch dimensions were then placed, securely ligated using ligature wires, and cinched back distal to the second molars. The appliances were mounted on the 3D models using the Rhinoceros software. Three different FFAs were simulated in this study:

1. The FFRD was attached from the maxillary first molar band to the archwire distal to the mandibular arch. A total force of 1.96 N [21] was simulated in this scenario, strictly corresponding to the standard force levels verified in established clinical protocols and manufacturer specifications (Figure 1A).
2. The PowerScope2 appliance was attached to the arch wires between the second premolar and first molar teeth on the maxilla, and between the canine and first premolar teeth on the mandible. A force of 2.54 N [22] was applied in this scenario, calibrated based on the manufacturer’s recommended baseline activation profiles (Figure 1B).
3. The metallic cap-splinted Herbst appliance was attached on the 3D model by using the Boolean method (Rhinoceros software). The length of the telescopic piston mechanism of the Herbst appliance provided an anterior position for the mandible by approximately one premolar tooth size (7 mm). This position stretched the muscles of mastication, which created a retrusive force on the mandible. To ensure a lifelike simulation result, several masticatory muscle combinations were installed into the finite element model and these combinations were reanalyzed. Ultimately, the analysis including only the posterior fibers of the temporalis muscle simulated a pragmatic simplifying assumption for this static model to reflect the expected clinical resistance vectors, resulting in a baseline force of 2.94 N derived from typical physiological soft tissue boundaries [13,23] (Figure 1C).

The geometric models were meshed to increase the accuracy of the finite element analysis (VRMesh software version 6.5, Virtual Grid Inc., Seattle, WA, USA). To confirm that the results were independent of the mesh density, a local mesh refinement analysis was performed across three distinct levels of element density (Coarse, Medium, and Fine). The maximum peak von Mises stress within the cortical bone surrounding the maxillary first molar was selected as the target parameter for comparison. By reducing the local element size from 0.4 mm in the medium mesh to 0.2 mm in the final fine mesh, the variance in peak stress stabilized at 2.8%. This minor variation fell safely below the 5% threshold, confirming mesh independence for all three simulated appliance configurations. The material elasticity modulus (Young’s modulus) and Poisson’s ratio [15] were then assigned to each structure in the models (via the Fempro interface of the Algor software, version 21 Algor Inc., Pittsburgh, PA, USA) (Table 1). In this study 8-node hexahedral elements were used. To obtain the most accurate results, the number of nodes and elements were increased at the regions to be analyzed (Table 2).

Regarding the interface conditions, fully bonded (tied) contact definitions were assigned to the tooth–bracket, tooth–PDL, PDL–bone, and appliance–splint boundaries to ensure structural continuity and ideal adaptation. For the FFRD and PowerScope2 setups, the archwires were structurally coupled within the bracket slots to simulate a securely cinched-back and ligated system without non-linear frictional sliding artifacts. To avoid the undesirable effects of the created forces, boundary conditions were applied by completely constraining the three translational degrees of freedom (UX = UY = UZ = 0) at the outermost nodes of the superior perimeters of the maxilla and zygomatic arch junctions, conforming to the natural mathematical formulations of the solid elements.

## 3. Results

The results of the von Mises stresses (MPa), principal stresses (MPa), and tooth displacement (mm) parameters were visualized using a color scale ranging from blue (lowest) to red (highest). The color blue indicates a lower amount of von Mises stress and tooth displacement, while the color red indicates higher values. Maximum and minimum principal stresses were interpreted in the same way. The nodes on the tip of the cusps and the apex of the roots were used to measure the tooth displacement for all posterior teeth; crown displacement was inherently calculated by taking the arithmetic average of the displacement values across all prominent cusp tips. For the anterior teeth, the displacement of the incisal edge nodes was directly utilized. For the sagittal plane measurements, a three-dimensional local coordinate system was used where negative values (−) indicate anterior (mesial) tooth displacement, while positive values (+) indicate posterior (distal) tooth displacement.

### 3.1. Amount of Tooth Displacement

**Figure 2:**
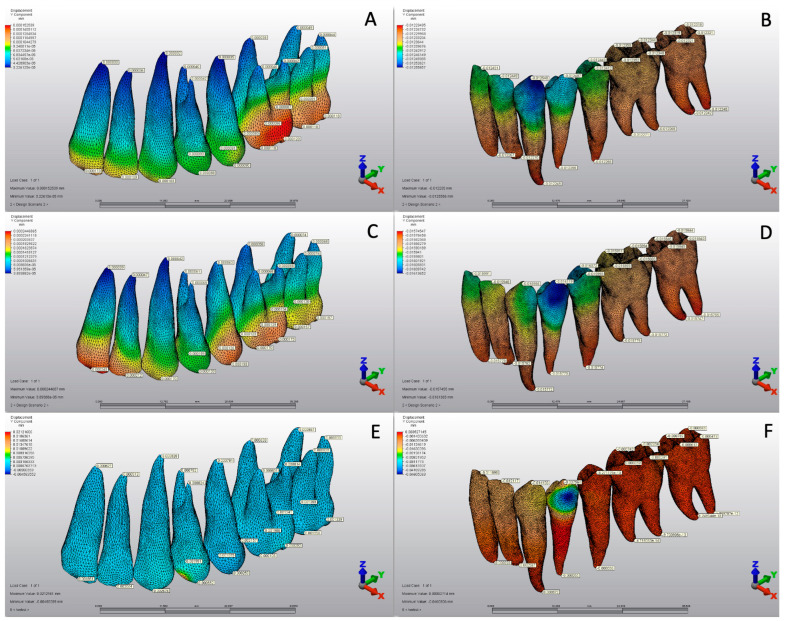
Tooth displacement values of the maxillary and mandibular arches. For the FFRD simulation: (**A**) displacement values of the maxillary teeth; (**B**) displacement values of the mandibular teeth. For the PowerScope2 simulation: (**C**) displacement values of the maxillary teeth; (**D**) displacement values of the mandibular teeth. For the Herbst simulation: (**E**) displacement values of the maxillary teeth; (**F**) displacement values of the mandibular teeth. Note: Different color scales and limits were utilized across the appliance simulations to maintain high visual contrast and allow a detailed inspection of localized displacement distribution patterns within each specific group.

Across all simulations, the maxilla exhibited distal tooth displacement, whereas mesial displacement occurred in the mandible (Table 3 and Table 4; Figure 2A–F). Notably, the magnitude of tooth movement was consistently greater in the mandible than in the maxilla.

In the maxillary arch, the extent of distal tooth displacement was comparable in the FFRD and PowerScope2 simulations (Figure 2A,C). However, the greatest amount of displacement was observed in the Herbst simulation (Figure 2E).

In the mandibular arch, the greatest amount of mesial tooth displacement was observed in the PowerScope2 simulation (Figure 2D), whereas the Herbst simulation demonstrated the least amount of displacement (Figure 2F). In addition, the crowns of the evaluated teeth exhibited greater displacement than their apices in all simulations (Table 3 and Table 4).

### 3.2. Von Mises Stress

**Figure 3:**
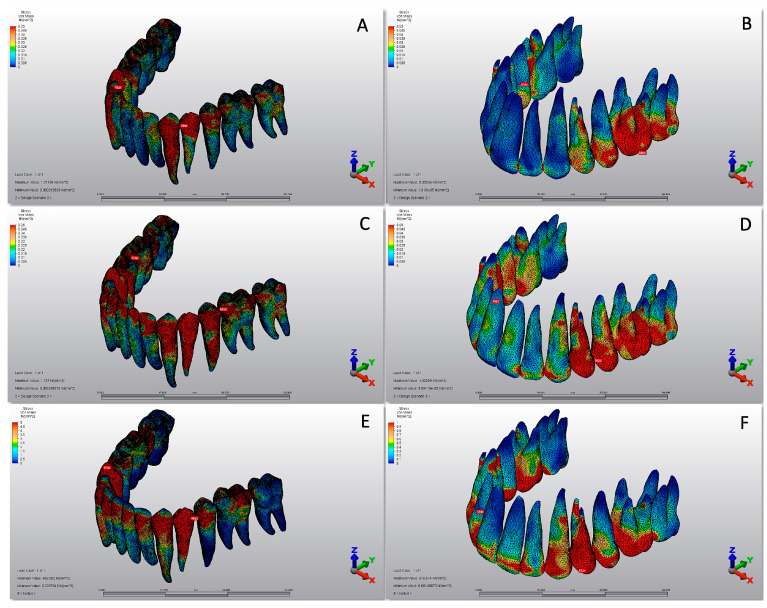
Von Mises stress distribution of the teeth after force application. For the FFRD simulation: (**A**) stress distribution of the mandibular teeth; (**B**) stress distribution of the maxillary teeth. For the PowerScope2 simulation: (**C**) stress distribution of the mandibular teeth; (**D**) stress distribution of the maxillary teeth. For the Herbst simulation: (**E**) stress distribution of the mandibular teeth; (**F**) stress distribution of the maxillary teeth. Note: Different color scales and limits were utilized across the appliance simulations to maintain high visual contrast and allow a detailed inspection of localized stress distribution patterns within each specific group.

In all simulated appliances in this study, the maxillary first molars exhibited the widest distribution of von Mises stress on the buccal surfaces (Figure 3B,D,F). In addition to these stresses, in the PowerScope2 simulation, the greatest stress distribution was observed on the buccal surfaces of the maxillary first and second premolars, while in the Herbst simulation, a broad stress distribution was detected on the buccal surface of the maxillary first premolar.

Regarding the mandibular dentition, the FFRD appliance demonstrated the highest stress distribution on the buccal surface of the mandibular canine (Figure 3A) (with a peak value of 1.271 MPa at the canine-premolar contact site, and 0.339 MPa on the maxillary first premolar crown). In the PowerScope2 model, elevated stress distribution was observed on the mandibular first premolar, second premolar, and canine teeth (Figure 3C), (with a peak value of 1.737 MPa at the second premolar–first molar contact site). In the Herbst model, increased stress was found on the mandibular first premolar and canine (Figure 3E) (with peak values of 432.092 MPa at the lower premolar contact site and 66.3 MPa on the upper first premolar cusp). Additionally, the incisal edges of a relatively small area of the lower incisors exhibited concentrated stress in the Herbst simulation.

Von Mises stress formed more intensely on the buccal surface of the relevant teeth in all simulations. However, the palatal surface of the upper second premolar and first molar crowns revealed increased stress areas in the Herbst simulation.

### 3.3. Principal Stress

**Figure 4:**
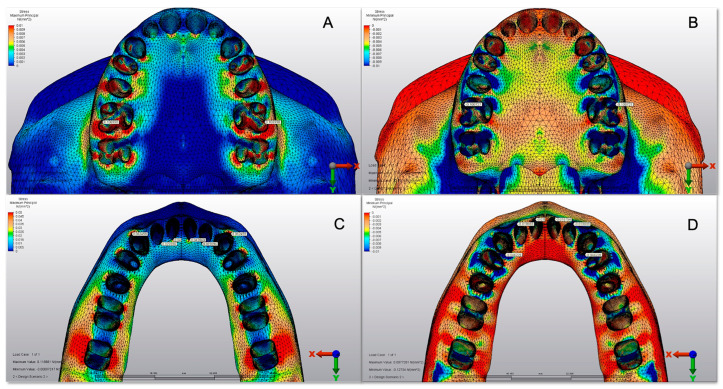
Occlusal view of the maximum and minimum principal stresses developed by the FFRD appliance: (**A**) maximum principal stress of the maxilla; (**B**) minimum principal stress of the maxilla; (**C**) maximum principal stress of the mandible; (**D**) minimum principal stress of the mandible. Note: Different color scales and limits were utilized across the appliance simulations to maintain high visual contrast and allow a detailed inspection of localized stress distribution patterns within each specific group.

**Figure 5:**
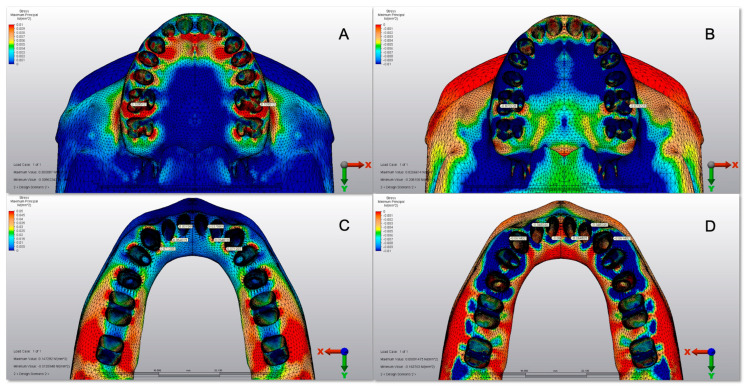
Occlusal view of the maximum and minimum principal stresses developed by the PowerScope2 appliance: (**A**) maximum principal stress of the maxilla; (**B**) minimum principal stress of the maxilla; (**C**) maximum principal stress of the mandible; (**D**) minimum principal stress of the mandible. Note: Different color scales and limits were utilized across the appliance simulations to maintain high visual contrast and allow a detailed inspection of localized stress distribution patterns within each specific group.

The maximum principal stress distribution was similar in the FFRD and PowerScope2 simulations (Figure 4 and Figure 5), revealing peak values of approximately 0.01 MPa on the maxilla and 0.05 MPa on the mandible. In the mandible, the principal stress was broadly distributed over the buccal and lingual alveolar regions of the second molars and gradually decreased toward the anterior teeth. A similar pattern was observed in the maxilla in the Herbst simulation; however, the PowerScope2 simulation showed a wider stress distribution in the palatal rugae region.

**Figure 6:**
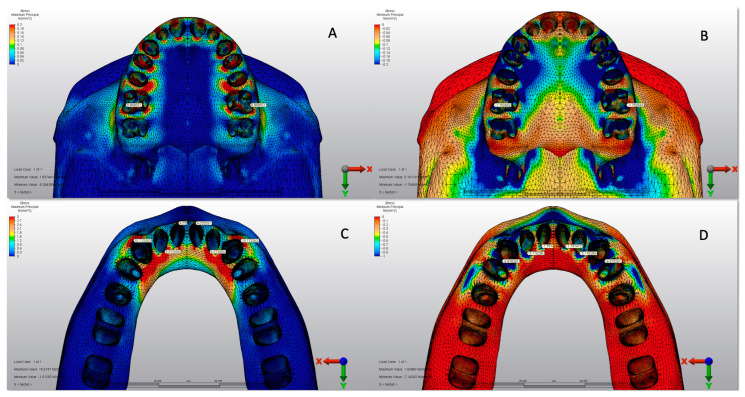
Occlusal view of the maximum and minimum principal stresses developed by the Herbst appliance: (**A**) maximum principal stress of the maxilla; (**B**) minimum principal stress of the maxilla; (**C**) maximum principal stress of the mandible; (**D**) minimum principal stress of the mandible. Note: Different color scales and limits were utilized across the appliance simulations to maintain high visual contrast and allow a detailed inspection of localized stress distribution patterns within each specific group.

The Herbst simulation exhibited the highest maximum and minimum principal stress values (reaching peaks of approximately 0.2 MPa on the maxilla and 3 MPa on the mandible). In the maxilla, stress was concentrated in the distobuccal and distopalatal alveolar regions of all posterior teeth except the second molars, whereas in the mandible, it was concentrated in the buccal and lingual alveolar regions of the canine teeth (Figure 6).

In all three simulations, the minimum principal stress was generally located opposite the regions of maximum stress in the maxilla (Figure 4, Figure 5 and Figure 6). In the Herbst simulation, it was concentrated on a small area of the buccal alveolus of the mandibular incisors and canines. In the PowerScope2 simulation, stress was distributed over a broad alveolar surface surrounding the incisors, canines, and premolars, whereas in the FFRD simulation, it was mainly observed in the distobuccal alveolus of the mandibular lateral incisor, canine, and first premolar.

## 4. Discussion

Although various FFAs have been used to correct Class II malocclusion, there is still a need for appliances that produce greater skeletal effects and fewer dentoalveolar effects, making this topic both current and clinically relevant. The appliances simulated in this study each offer specific advantages and are widely used in clinical practice. However, there is a notable lack of studies comparing their mechanical effects using the finite element method. In addition, comprehensive orthodontic data on detailed three-dimensional simulations of these appliances are still limited. Therefore, this study was designed to model, simulate, and compare these appliances.

The finite element method has been widely used in several studies to evaluate the effects of functional orthodontic appliances [11,12,13,14,15,24,25]. However, the existing literature remains fragmented, as most studies have addressed isolated components of the treatment response rather than its comprehensive biomechanical impact. Using this approach, Ulusoy and Darendeliler [13], Gupta et al. [15], and Song et al. [24] examined the stress distributions in the masticatory muscles, ligaments, and associated bony structures under various orthopedic appliances. Similarly, Chaudry et al. [11] focused on the stress alterations in the mandibular teeth, condyle, and alveolar bone under resting and load-bearing conditions, while Zadi et al. [12] evaluated the mandibular response to the Herbst appliance using a viscoelastic finite element model. Although these studies provide valuable insights, their scope is limited to specific anatomical sites or mechanical outcomes; furthermore, they have not assessed the overall effects of these appliances. Panigrahi and Vineeth [14] performed a more extensive analysis by assessing the tooth displacement and stress distribution in the condyle and alveolar bone under a 200 g force; however, their model did not incorporate an actual orthopedic appliance, raising uncertainty regarding the clinical relevance of the simulated force application. Therefore, previous finite element studies have provided a limited biomechanical representation of functional appliances. In this context, the present study provides a more comprehensive evaluation by simulating three different FFAs and comparing their mechanical effects using finite element analysis.

The accuracy and reliability of a finite element analysis can be enhanced when the model includes more elements and nodes [26]. Yu et al. [27] constructed a finite element model of the entire skull and maxilla which included 22,236 elements and 71,714 nodes. Panigrahi and Vineeth [14] used 13,590 elements and 18,582 nodes, and Gautam et al. [28] used 108,799 elements and 193,633 nodes. In this study, we modelled the FFRD, PowerScope2, and Herbst simulations using 229,475 nodes and 1,010,391 elements; 226,194 nodes and 992,453 elements; and 210,079 nodes and 969,781 elements, respectively. As such, the dentoalveolar structures and oral conditions were simulated in a more realistic manner in this study.

While constructing the skull we used a smaller number of elements in the inner parts of the anatomical structures compared to the parts that would be analyzed for the study. Moreover, instead of relying on a completely uniform global mesh layout, we selectively elevated the element and node density in steep, narrow, and highly complex stress zones. This localized refinement protocol allowed us to maintain an optimized, uniform meshwork and eliminate potential mathematical inaccuracies or artificial structural stiffening without altering the fundamental element formulations. Through this modelling technique we aimed to create a high-quality meshwork, to avoid a complex analysis and to enhance the reliability of the mathematical calculations.

The greatest mesial displacement of the mandibular incisors was observed in the PowerScope2 simulation, whereas the Herbst and FFRD simulations produced smaller and comparable amounts of displacement. Similarly, Arora et al. [29] who compared the effects of the FFRD and the PowerScope appliances in a clinical examination, reported a greater increase in the mandibular plane–incisor plane angle in patients treated with PowerScope, which is consistent with our findings. The mesial displacement of the posterior teeth was also greatest in the PowerScope2 simulation and least in the Herbst simulation. In line with this, Moro et al. [30] evaluated rigid, flexible, and hybrid FFAs and found that the rigid appliances produced greater skeletal retention than the flexible and hybrid designs. Accordingly, the minimal mesial displacement observed in the Herbst simulation in our study within the constraints of our muscle force assumptions indicates that this rigid design produces significantly less initial mandibular dentoalveolar displacement than the other appliances under these specific static conditions. While this theoretically suggests a different biomechanical pattern that may favor anchorage preservation, it should not be interpreted as direct evidence of a greater clinical skeletal adaptation or condylar remodeling. In clinical reality, the long-term skeletal and dentoalveolar outcomes are heavily influenced by confounding variables such as the patient’s growth status, total treatment duration, archwire sequencing, and bracket positioning, which cannot be captured in a short-term simulation In addition, the total maxillary distal displacement was greatest in the Herbst simulation and lowest in the FFRD simulation. The Herbst appliance also produced the highest degree of incisor displacement in the distal direction. This headgear-like effect may play an important role in reducing excessive overjet and improving the correction of Class II molar relationships in clinical settings.

The magnitude of the applied force is a key determinant of the biomechanical response in functional jaw orthopedics. It is important to note that the applied forces differed among the groups (1.96 N for the FFRD, 2.54 N for the PowerScope2, and 2.94 N for the Herbst) to reflect the realistic clinical and manufacturer-recommended baselines of each appliance. Additionally, the Herbst appliance model incorporated distinct biological variables, such as the simulation of the posterior fibers of the temporalis muscle, representing three distinct mechanical systems rather than isolated appliance designs. Consequently, the higher von Mises and principal stress values observed in the Herbst group are partially a reflection of this higher force magnitude and the muscle load vectors. However, the vector and specific localization of stress concentrations—rather than just the absolute peak values—highlight the distinct mechanical behavior dictated by each appliance design. Nevertheless, direct comparisons of absolute stress magnitudes between the appliances should be interpreted with caution due to these varying force baselines. Furthermore, our models were only partially validated against the trends reported in the literature, and a direct patient-specific clinical validation was not performed. Therefore, these absolute numerical results may not directly reflect complex in vivo clinical outcomes.

This study also compared the FFRD simulation with the functional appliance model evaluated by Panigrahi and Vineeth [14], as both studies applied comparable force magnitudes. However, in contrast to their findings, we observed less individual tooth displacement in the maxilla and greater tooth displacement in the mandible. In the finite element analyses, even when similar force magnitudes are applied, differences in the force vector orientation and appliance configuration may lead to distinct dentoalveolar displacement patterns. Therefore, the discrepancy between the two studies may not be solely related to appliance mechanics but may also reflect differences in the biomechanical response within the craniofacial complex under similar loading conditions.

In the present study, the von Mises stress values exhibited a direct relationship with the magnitude of the applied force, with the highest amount of stress concentrations recorded in the Herbst appliance simulation. Across all loading conditions, the greater stress was primarily localized around the point of force application and progressively attenuated with increasing distance from the region of the load source, resulting in a broader yet more superficial stress distribution pattern on the tooth and alveolar surfaces. This biomechanical response is consistent with the observed tooth displacement patterns and suggests that the stress transmission characteristics of the appliance may play a determining role in the regional distribution of orthodontic forces. Chaudhry et al. [11] reported that the von Mises stress in a patient treated with the Forsus appliance increased in parallel with an increasing force magnitude, which suggests that the force level is a critical determinant of stress distribution.

The PowerScope2 appliance produced a broader von Mises stress distribution across both arches in this study, whereas the FFRD showed a more localized stress pattern, particularly in the maxilla. The Herbst appliance demonstrated highly stressed regions at the incisal edges of the maxillary incisors. Although the teeth were connected to each other by an archwire, the FFRD generated minimal stress on the maxillary incisors beyond the incisal contacts. In contrast, the PowerScope2 showed a more uniform stress pattern at the crown and root surfaces of the maxillary incisors, suggesting a more parallel and homogeneous distalization effect. The elevated stress across incisal regions observed with the Herbst appliance may also indicate some distal tipping of the incisors with this appliance.

In the mandible, both the magnitude of the von Mises stress and the distribution of high-stress regions were greater in the PowerScope2 and Herbst simulations than in the FFRD simulation (Figure 3). This finding supports the above-mentioned argument of the present study, indicating that the greatest amount of mesial tooth displacement in the mandible occurred in the PowerScope2 simulation. Moreover, this result aligns with the clinical findings of Shetty et al. [31], who reported a greater dentoalveolar effect with the PowerScope appliance compared with the FFRD. While this clinical study helps contextualize our mechanical findings, the alignment should be interpreted cautiously; our simulation evaluates the initial biomechanical stress, whereas clinical outcomes are driven by long-term biological remodeling and patient-specific anatomy.

A balanced stress distribution of the buccal and palatal alveoli exhibits a parallel displacement of the posterior teeth. Regarding the maxillary posterior region, while the Herbst and PowerScope2 simulations displayed an almost balanced buccal-palatal stress distribution, the FFRD exhibited stressed areas only on the buccal alveolus surface. This difference reveals that the buccal crown tipping of the posterior teeth is possible with the FFRD.

In this study, similar to the von Mises stress findings, the maximum principal stress in the mandible was observed in the Herbst simulation in both jaws. In the maxilla, the maximum principal stress was consistently localized on the distal surface of the tooth socket in all simulations (Figure 4A, Figure 5A and Figure 6A). In addition, a stress concentration over a broad palatal area of the incisors was observed in the PowerScope2 simulation. Although this distribution was unexpected in the Herbst appliance, there is no orthodontic literature specifically evaluating Herbst-appliance–induced stress patterns to allow a direct comparison with our findings.

In the mandible, the maximum principal stress was predominantly concentrated on the buccal and lingual alveolar surfaces of the posterior teeth in the FFRD and PowerScope2 simulations, whereas in the Herbst model, the stress was mainly localized on the lingual surface of the anterior teeth. These results suggest that the semi-rigid FFAs chiefly transfer loading stress to the posterior dentoalveolar region in the mandible. The observed stress distribution may be attributable to the cinch-back of the mandibular archwire distal to the second molars, which likely alters load transfer characteristics and promotes stress concentration in the posterior segment.

## 5. Limitations

The numerical data obtained from the finite element analyses cannot reflect the long-term biological and clinical changes in a living tissue [32]. Instead, the initial stress and displacement values evaluated in the current study provide a standardized platform to compare the mechanical behavior of the simulated appliances.

This study has some limitations, particularly regarding the force configuration in the Herbst simulation. Ideally, functional appliances induce an anterior mandibular advancement. In our preliminary multi-muscle simulations including the masseter and medial pterygoid muscles, the heavy vertical and protrusive vectors of these muscle groups counteracted the telescopic piston mechanism in a static finite element analysis environment, causing an artificial backward distortion of the mandibular teeth. Since such vectors do not represent the clinical action of a fixed functional appliance, a series of preliminary boundary and force configurations were evaluated to isolate the true structural resistance. Biomechanically, the posterior fibers of the temporalis muscle provide the primary horizontal retrusive vector when the mandible is held forward. Therefore, isolating this specific muscle group was selected as a pragmatic simplifying assumption for this static model to simulate the clinical biomechanical resistance (2.94 N).

However, because a quantitative sensitivity analysis was not performed, it is critical to recognize that the Herbst outcomes depend heavily on this specific baseline configuration, and these findings should be interpreted with caution as they represent a simplified abstraction of complex in vivo muscle dynamics rather than a uniquely validated representation.

Regarding the anatomic structures, the PDL was modeled with a uniform thickness of 0.2 mm and linear elastic properties. In real clinical situations, the PDL exhibits complex non-linear behavior, viscoelastic properties, and regional thickness variations across different root regions. While this uniform, linear simplification is a common baseline approach in dental finite element analysis literature, it might affect the absolute stress values on the teeth. Therefore, our results should be evaluated as a relative comparison of mechanical tendencies between the systems rather than absolute physical or biomechanical values.

Another limitation of this study is the use of a single patient-derived model. Individual variations in craniofacial morphology, root anatomy, cortical bone thickness, PDL morphology, dental arch form, and occlusal relationships may alter the displacement and stress distribution patterns. Therefore, the present findings do not establish the clinical superiority of one appliance over another and should be validated by further finite element analyses using different anatomical models and by clinical studies.

Additionally, this finite element model provides a static representation of initial force application and does not fully account for clinical factors that dictate long-term success. Crucial parameters such as active biological bone remodeling, variation in anchorage designs, patient-specific anatomical diversity, and patient compliance are not represented in the present simulation. Therefore, while our data offer valuable theoretical comparisons, this manuscript explicitly avoids implying that the present numerical simulation confirms or predicts absolute clinical effectiveness.

In conclusion, within the inherent limitations of this numerical simulation—such as its static nature, single-model design, uniform PDL simplification, and lack of direct clinical validation—these initial biomechanical trends should be interpreted with caution and serve as a baseline for future dynamic clinical investigations rather than definitive clinical outcomes.

## 6. Conclusions

Within the limitations of this preliminary, patient-specific finite element study, the results can be summarized as follows:

1. The Herbst simulation generated the greatest amount of maxillary distalization, including the distal displacement of the maxillary incisors, suggesting a more pronounced simulated headgear-like effect within this model geometry compared with the FFRD and PowerScope2 simulations.
2. The PowerScope2 simulation demonstrated the greatest mesial displacement of the mandibular teeth, which may be consistent with the previously reported dentoalveolar tendencies of semi-rigid functional appliances. In contrast, the Herbst simulation resulted in the least mesial displacement of the mandibular teeth, highlighting a distinct displacement pattern characterized by a reduction in the simulated mandibular dentoalveolar movement within this specific model geometry. The higher von Mises and principal stress values observed in the Herbst simulation may indicate regions of increased mechanical demand within the appliance components and supporting structures under the simulated loading conditions. However, the relationship between these stress concentrations and clinical complications, such as cementation failure, welding site fracture, or deformation of the telescopic mechanism, requires further experimental and clinical validation.
3. In the maxillary posterior region, the FFRD showed predominantly buccal alveolar stress concentration areas, suggesting a potential tendency for buccal crown tipping of the posterior teeth.
4. The semi-rigid functional appliances mainly transferred stress to the mandibular posterior dentoalveolar region, whereas the Herbst appliance produced more pronounced stress concentration in the anterior mandibular alveolar region.

Overall, the findings of this finite element analysis study revealed that the Herbst simulation was associated with greater maxillary distal displacement and reduced mandibular dentoalveolar displacement compared with the FFRD and PowerScope2 simulations. Because this model strictly assesses instantaneous physical displacements rather than biological skeletal adaptation, these results must be interpreted solely as model-specific biomechanical tendencies rather than direct evidence of clinical effectiveness or skeletal changes. The patient-specific anatomical and occlusal variations may influence the displacement and stress patterns of the analysis. Therefore, further finite element analyses using multiple patient-derived models, as well as prospective clinical studies, are required to confirm the clinical relevance of our findings.

## Figures and Tables

**Table 1:** Young’s modulus and Poisson’s ratio for the materials used in the study.

*Material*	*Young’s Modulus* (MPa)	*Poisson’s Ratio*
Cortical bone	13,700	0.3
Trabecular bone	7900	0.3
Teeth	20,290	0.3
Periodontal ligament	7	0.49
Orthodontic archwire	200,000	0.3
FFRD	200,000	0.3
Herbst	200,000	0.3
PowerScope2	200,000	0.3

**Table 2:** The number of elements and nodes used in the 3D models.

*Appliance Model*	Number of Elements	Number of Nodes
FFRD	1,010,391	229,475
PowerScope2	992,453	226,194
Herbst	969,781	210,079

**Table 3:** Displacement values of the maxillary teeth under different fixed functional appliance simulations.

Tooth	Selected Nodes	Displacement Values (mm)		
		FFRD	PowerScope2	Herbst
Central incisor	Incisal margin	0.00011	0.00024	0.00185
	Apex	0.00003	0.00004	0.00063
Lateral incisor	Incisal margin	0.00011	0.00021	0.00206
	Apex	0.00004	0.00005	0.00091
Canine	Incisal margin	0.00010	0.00017	0.00268
	Apex	0.00003	0.00004	0.00082
First premolar	Occlusal margin	0.00009	0.00015	0.00127
	Apex	0.00004	0.00006	0.00081
Second premolar	Occlusal margin	0.00009	0.00016	0.00066
	Apex	0.00004	0.00006	0.00078
First molar	Occlusal margin	0.00010	0.00015	0.00110
	Apex	0.00004	0.00006	0.00076
Second molar	Occlusal margin	0.00010	0.00015	0.00128
	Apex	0.00005	0.00007	0.00087

Note: In the coordinate system, positive values (+) indicate posterior (distal) displacement, while negative values (−) indicate anterior (mesial) displacement.

**Table 4:** Displacement values of the mandibular teeth under different fixed functional appliance simulations.

Tooth	Selected Nodes	Displacement Values (mm)		
		FFRD	PowerScope2	Herbst
Central incisor	Incisal margin	−0.01243	−0.01599	−0.01189
	Apex	−0.01227	−0.01578	0.00022
Lateral incisor	Incisal margin	−0.01245	−0.01595	−0.01212
	Apex	−0.01227	−0.01578	0.00059
Canine	Incisal margin	−0.01255	−0.01596	−0.01419
	Apex	−0.01225	−0.01577	0.000372
First premolar	Occlusal margin	−0.01249	−0.01612	−0.00756
	Apex	−0.01226	−0.01577	−0.00026
Second premolar	Occlusal margin	−0.01241	−0.01599	−0.00000
	Apex	−0.01226	−0.01577	−0.00009
First molar	Occlusal margin	−0.01235	−0.01590	−0.00084
	Apex	−0.01227	−0.01578	−0.00000
Second molar	Occlusal margin	−0.01232	−0.01584	−0.00058
	Apex	−0.01225	−0.01575	−0.00000

Note: In the coordinate system, negative values (−) indicate anterior (mesial) displacement, while positive values (+) indicate posterior (distal) displacement.

## Data Availability

The datasets generated and/or analyzed during the current study are not publicly available but are available from the corresponding author on reasonable request.

## Abbreviations

FFRD	Forsus^TM^ Fatigue Resistant Device
FFAs	Fixed functional appliances
CBCT	Cone–beam computerized tomography
STL	Standard Tessellation Language
MPa	Megapascal
